# Cardiometabolic Syndrome and Increased Risk of Heart Failure

**DOI:** 10.1007/s11897-016-0298-4

**Published:** 2016-08-18

**Authors:** Helene von Bibra, Walter Paulus, Martin St. John Sutton

**Affiliations:** 1 Clinic for Endocrinology, Diabetes & Vascular Medicine, Klinikum Bogenhausen, Städt. Klinikum München GmbH, Munich, Germany; 2 Institute for Cardiovascular Research Vrije Universiteit, VU University Medical Center Amsterdam, Amsterdam, The Netherlands; 3 Department of Medicine, Cardiovascular Division, University of Pennsylvania, Philadelphia, PA USA

**Keywords:** Metabolic syndrome, HFPEF, Insulin resistance, Diastolic dysfunction, Carbohydrate restriction

## Abstract

Approximately 50 % of patients with heart failure have diastolic heart failure (HFPEF) with the major predisposing risk factors age, inactivity, obesity, insulin resistance (IR), type-2 diabetes, and hypertension. The prognosis of HFPEF is comparable to that of systolic heart failure, but without any specific or effective treatment. This review presents a biomathematically corrected diagnostic approach for quantification of diastolic dysfunction (DD) via the age dependency of diastolic function. Pathophysiological mechanisms for DD in the cardiometabolic syndrome (CMS) are mainly based on downstream effects of IR including insufficient myocardial energy supply. The second section discusses therapeutic strategies for the control and therapy of CMS, IR, and the associated DD/HFPEF with a focus on dietary therapy that is independent of weight loss but improves all manifestations of the CMS and reduces cardiovascular risk.

## Introduction

The cardiometabolic syndrome (CMS), also known as insulin resistance (IR) syndrome [1], is a combination of primarily metabolic disorders with IR as the underlying disorder. Its strong link with cardiovascular disease makes CMS the leading cause of morbidity and mortality in the Western world [2, 3].

However, the perilous consequences of CMS are not only coronary artery disease (CAD) but also non-ischemic myocardial dysfunction and heart failure (HF) [4] associated with obesity cardiomyopathy, insulin resistance cardiomyopathy, and diabetes cardiomyopathy [5–7] which suggest that IR is the underlying disorder [8]. Indeed, IR and HF are strongly correlated [9–13] with causal associations between IR and cardiac dysfunction that are observed in the development of IR by genetic and also by environmental factors [14]. In addition, HF predisposes to the development of cardiac and/or systemic IR and type-2-diabetes [15].

The reciprocal relationship between IR and HF is important because it implies therapeutic potential for HFPEF and its predecessor diastolic dysfunction (DD): prevalence and prognosis of HFPEF equal those of systolic HF [4, 16–18] but therapeutic studies have been unsuccessful [19, 20••, 21••]. This review discusses underlying pathophysiological mechanisms of DD/HF associated with CMS, a potential solution to diagnostic problems and the resulting options for therapy.

### CMS and DD/HFPEF

#### Background and Prevalence of CMS

CMS has been defined by the World Health Organization and the National Cholesterol Education Program Adult Treatment Panel III with central obesity and IR as key risks [22••]. The diagnosis requires the presence of any three out of these factors: hyperglycemia (as ultimate result of IR), hypertension, central obesity, dyslipoproteinemia (high triglyceride and/or low high-density lipoprotein (HDL) levels). Age contributes to an increased prevalence of CMS so that an epidemic increase in CMS and its downstream effects on CAD, stroke, and HF imply an ominous impact worldwide [22••]. Approximately 34 % of the adult US population and about 4 % of the adolescents have CMS that is most frequently driven by obesity. The prevalence of CMS is estimated at 15–25 % in European adults and ranges in the Middle East from 20–37 % in the Gulf States to 28–39 % in Turkey or in urban populations of India [22••].

#### Prevalence and Clinical Signs of DD/HFPEF

Over the last two decades, HFPEF has become an increasingly important problem. Prevalence of its preceding abnormality DD is high, ranging from 11 to 27 % in the general adult population, but with a mean of 35 % in individuals with CMS, 12, 35, and 45 % in increasing severity of obesity and 50–70 % in pre-diabetes and in type-2 diabetes [8, 20••, 23]. However, the triggers for the progressive transition into clinical HF remain to be clarified [20••].

The typical clinical symptoms are exertional dyspnea and decreased exercise capacity, leading to reduced quality of life especially in the elderly. If diastolic function at rest is normal, diastolic reserve should be assessed and a 6-min walking test scheduled. In spite of the known risk factors overweight, IR and type-2 diabetes, DD is under diagnosed in these individuals who often conceal their reduced physical performance or have scaled down their activities.

#### Pathophysiological Mechanisms in CMS Associated Risk for HF

A fundamental principle is known from the ischemic cascade: insufficient energy/oxygen supply initially impairs myocardial diastolic function and only subsequently results in systolic dysfunction [24]. This immediate effect is reversible and without any changes of myocardial structure.

Accordingly, a two-step model appears meaningful for HF and the preceding DD associated with CMS: Its risk factors obesity, IR, type-2 diabetes, dyslipoproteinemia, and hypertension metabolically induce insufficient energy supply, intermittently, repetitively, and reversibly [23, 25, 26•]. In a second step, remodeling mechanisms increase myocardial stiffness and impair late diastolic function. An early and potentially treatable cause such as IR may be differentiated from later remodeling so that therapeutic interventions would not any more target myocardial stiffness alone but focus on earlier metabolic factors in favor of potential prevention.

Overweight impacts on more than half the adult population leading most often to IR. This applies also to cardiology patients and implies metabolic constraints for myocardial energy supply in addition to the individual cardiac disease [8, 25, 27]. Recently, IR was confirmed as one of the three top risk factors for DD in non-diabetic individuals and the top risk factor in type-2 diabetes [21••].

#### Insulin Signaling Links to Cardiac Function

Cardiac IR may develop independently of systemic IR. More often and subsequent to prolonged or excessive overnutrition, systemic IR relevantly contributes to secondary cardiac IR with oxidative stress and neurohumoral/sympathetic and/or cytokine imbalance. Two different pathways of insulin signaling impact on cardiac function [13]:

- The phosphatidylinositol-3 kinase/protein kinase B (Akt) signaling pathway elicits mainly beneficial metabolic responses. The downstream mechanisms include the activation of Akt and atypical protein kinase-C isoforms mediating the following reactions: Glucose transporter type-4 (Glut4) translocation leading to glucose uptake in myocardial/muscular tissue, nitric oxide mediated coronary dilatation, metabolic substrate flexibility and energy homeostasis, and balanced calcium handling.
- The mitogen-activated protein kinase (MAPK) signaling pathway elicits growth factor-like responses that contribute to growth and remodeling responses such as myocellular hypertrophy, cardiac fibrosis, impaired myocardial-endothelial signaling and death of myocardial and endothelial cells.

**Fig. 1:**
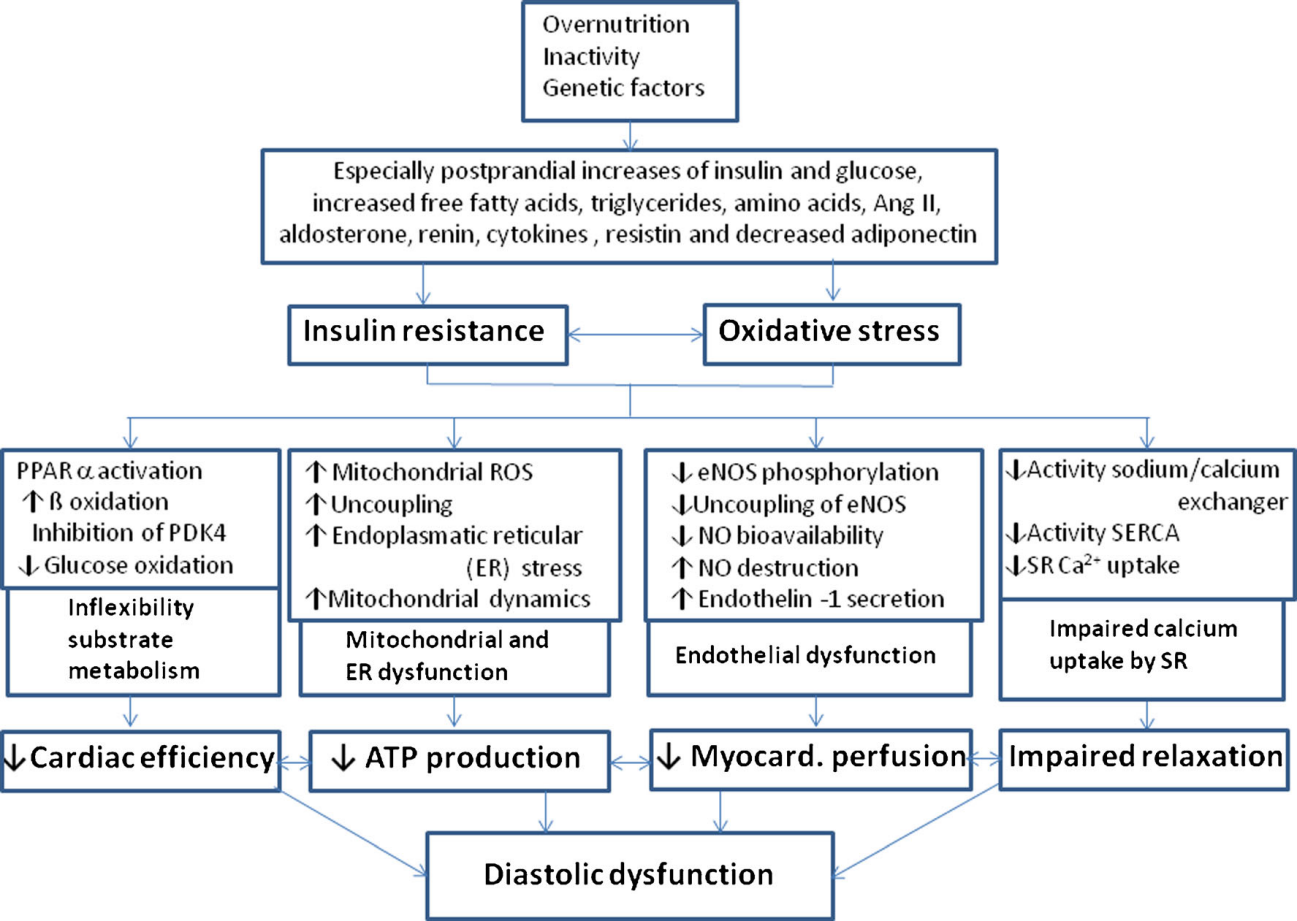
Molecular mechanisms in cardiac insulin resistance via overnutrition and impaired insulin signaling leading to repetitive intermittent lack of energy in the early stage of cardiac dysfunction that is DD. The progression to remodeling processes including myocellular hypertrophy, altered titin, collagen and fibrosis metabolism, accumulation of triglycrides, and/or advanced glycemic end products and also the activation of the renin-angiotensin and sympathetic nervous system will lead to further myocardial cell damage and, potentially, to contractile dysfunction and/or heart failure. *Ang II* angiotensin II, *ROS* reactive oxygen species, *ER* endoplasmatic reticulum, *eNOS* endothelial nitric oxide synthase, *SERCA* sarcoplasmic endoplasmic reticulum Ca2+ - ATPase 2a, *SR* sarcoplasmatic reticulum (modified from [13])

The resultant complex and reciprocal metabolic mechanisms involve altered substrate use and decreased myocardial ATP generation, endothelium induced dysregulation of myocardial perfusion and impaired calcium handling that lead to DD in its early stage (Fig. 1) before inflammatory and cytokine abnormalities, lipo- and glucotoxicity, oxidative stress from reactive oxygen species, upregulation of the renin-angiotensin-aldosterone (RAAS) and sympathetic nervous system induce remodeling processes. These imply myocellular hypertrophy, fibrosis, collagen, and titin modifications and accumulation of triglycerides and/or advanced glycemic end products leading to cell damage and further deterioration of DD and also contractile function. The specific impact and sequence of these mechanisms for the development of HF in man remain to be clarified [13, 14, 19, 20••, 27, 28].

#### Diagnostic Criteria for the Risk of HF in CMS

HFPEF/DD is a functional diagnosis with many different comorbidities. The major risk factors are age and CMS that include obesity, IR, type-2 diabetes, and hypertension, furthermore CAD, anemia, chronic obstructive pulmonary disease, sleep apnea, renal disease, and rare congenital abnormalities. Given the epidemic of CMS, early accurate diagnosis of DD is of pivotal importance for the development of preventive strategies. In contrast to HFREF and systolic dysfunction, there is no consensus as to how to quantify DD [8, 20••, 25]. This problem contributes to the lack of clinically relevant results in therapeutic studies [16, 20••, 26•].

The European and American guidelines define DD as an abnormal LV filling pressure that may be estimated non-invasively by E/E’ >15. For the intermediate range 8–15, additional parameters of potential DD involve mainly increased left atrial volume or LV mass, a diminished mitral inflow ratio E/A and/or biomarkers [17]. These echocardiographic metrics were considered partly non-sensitive or inconclusive [21••, 29]. A single criterion for quantification of myocardial diastolic function is not available to assess DD although tissue Doppler derived E’ has prognostic potential and also correlates with exercise capacity [8, 30••, 31].

**Fig. 2:**
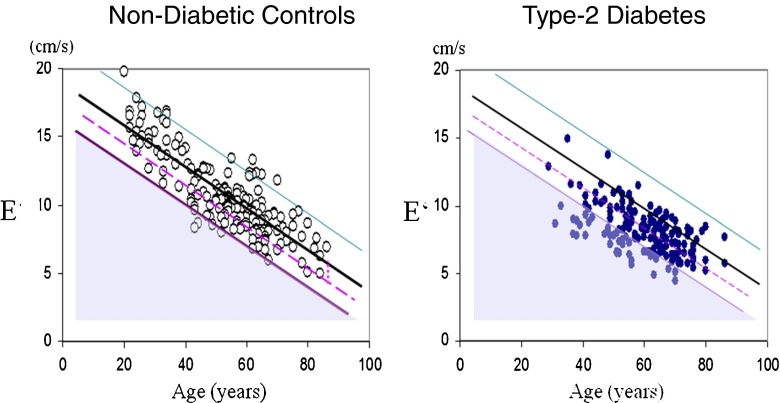
Diastolic myocardial function E’ as function of age in non-diabetic controls (*left*) and type-2 diabetes (*right*) with the regression line (*black*) and upper and lower tolerance intervals (*purple* and *green*) from healthy controls. Diastolic dysfunction is defined as difference E’ – E’_norm_ on the regression line >2.86 (*shaded triangle*) and risk for diastolic dysfunction if the deficit is >50 % of this cut off level (between the dashed line and the lower tolerance line) (modified from [21••])

A biomathematical approach may solve this diagnostic problem: age is such a dominant determinant of diastolic myocardial function that it accounts for 88 % predictability of diastolic function but only 39 % of systolic function [21••]. Accordingly, DD needs to be defined as the deficit between the E’ and an age-related normal value from healthy individuals E’_norm_ (Fig. 2). Consequently, risk factors for DD are a function of this deficit E’_norm_ – E’. This approach avoids the biomathematical error of defining risk factors that also depend heavily on age. Instead, the central importance of metabolic abnormality, i.e. IR, is revealed [21••].

### Non-Pharmacological Therapeutic Strategies for CMS

The epidemic of CMS is mainly caused by environmental factors such as inactivity and the availability of cheap and “fast” foods rich in energy from starches and fat. The single major cause of poor health in cardiology patients is poor diet.

However, doctors receive very little training in healthy nutrition in spite of reviews that actually remove the ban from fat and demonstrate the risks of high carbohydrate intake [32••, 33••, 34].

#### Dietary approaches

In CMS, dietary approaches aim for these issues: (1) weight normalization and maintenance long-term, (2) elimination of postprandial glucose and insulin peaks without weight loss, and (3) reduction of the risk factors dyslipoproteinemia and hypertension.

(1) Weight Reduction and Maintenance:

A relevant reduction of weight may abolish IR/hyperinsulinemia by the reduction of ectopic fat accumulation and improve all manifestations of the CMS [35]. It is achieved rather by diet than by exercise only [36]. The fundamental hypocaloric nutrition renders various diets suitable:

- *Low-fat* nutrition has been traditionally recommended as restriction of fat to about 60 g daily and high carbohydrate ingestion [34, 37•]. Via a daily energy deficit of 500 kcal, mean weight loss may be 3–4 kg within 6 months followed commonly by a regain in weight [38, 39]. There is no evidence for more effective weight loss compared to other hypocaloric diets [40] but evidence for a long-term increase of overweight and cardiovascular disease [32••, 41, 42].
- *Carbohydrate restricted diets* show superior weight loss by about 3 kg within 6 months if compared to low-fat diets [43, 44] with some subsequent regain, so that weight loss at 12 months is 1–2 kg greater but not significant [44].
- *Carbohydrate modification* implies a reduction of glycemic index (GI) and glycemic load (GL) (table) resulting effectively in weight loss [45••, 46, 47]. An example is the LOGI (low glycemic and insulinemic) method [48] based on recommendations for obesity therapy at the Boston children’s hospital [49] with avoidance of refined starch and saccharose products and preference for food with low GI (vegetable, legumes, and whole grain products) minimizing postprandial glucose and insulin peaks [46]. Meta-analysis demonstrates superior weight loss within 6 months for diets with reduced GI and/or GL including the Mediterranean diet [46, 47]. Additionally, the cardiovascular risk factors dyslipoproteinemia, hypertension [30••, 37•, 50, 51], and IR are improved [30••, 52].

Only 20 % of subjects maintain weight loss long-term. Attainment of this goal may be supported by two measures:

- A decrease of energy density to <125 kcal/100 g of consumed food is recommended given population based values of ≥160 kcal/100 g [34]. This aim requires to consume more food high in water and fibers (salad, vegetable, fruit, fish, milk/milk products, and lean meat) and less high-energy food (fast food rich in fat and sugar, customary bakery products, sugared lemonades, and fruit juices) [53]. An advisable low-energy meal may be a salad with breast of turkey and olive oil dressing in spite of its 40 % content in fat [48].
- Improved satiety is achieved by increasing water and fiber content of food and protein intake to a total of 30 % (about 2 g/kg body weight) [47, 54]. In healthy people, no harm has been shown for such a moderate increase of protein intake but advantages for maintaining weight loss [55].

(2) GL Restriction for Normalizing IR Without Weight Loss:

In the course of CMS, long-term normalization of weight is often unrealistic. Hence, dietary strategies are preferable that normalize postprandial insulin and glucose peaks and IR independent of weight loss in these high risk individuals [56].

Postprandial hyperglycemia subsequent to a carbohydrate meal is common in individuals with IR. The associated fluctuations of glucose and insulin are known cardiovascular risk factors [57, 58] depending mainly on two mechanisms:

- Reduced insulin sensitivity, i.e., IR leads to higher postprandial glucose peaks in these compared to insulin sensitive individuals [48, 59]. The more insulin resistant a person is, the more insulin is required to keep blood glucose within normal limits rendering the normalization of hyperinsulinemia/IR an important therapeutic target.
- Postprandial glucose values are directly determined by the quality, i.e. GI, and quantity, i.e. GL, of the consumed carbohydrates. Their specific reduction provides a practical tool to prevent postprandial hyperglycemia and hyperinsulinemia (Table 1) [57, 60]. In lean healthy individuals, 37 % of the postprandial glucose levels are determined by GI, but 90 % by GL that contributes also to a greater extent to atherogenic dyslipoproteinemia [32••, 61].

**Table 1:** Table of glycemic index (GI) and glycemic load (GL) in commonly used foods containing relevant amounts of carbohydrates

Food	GI/100 g	Serving Size (g)	Available Carbohydrates g/serving size	GL
Spaghetti, white boiled	44 ± 3	180	48	21
Spaghetti, full grain boiled	37 ± 5	180	42	16
Cornflakes	81 ± 3	60	52	42
Baguette	95 ± 15	60	30	30
Wheat bread	70 ± 0	60	28	20
Rye kernel bread	50 ± 4	60	24	12
Rice, long grain boiled	56 ± 2	150	41	23
Rice, brown boiled	55 ± 5	150	33	18
Potatoes baked	85 ± 12	150	30	26
Potatoes cooked	56 ± 101	150	17–26	11–18
Potato chips	54 ± 3	90	63	33
Peas	35 ± 4	150	15	5
Carots (raw and boiled)	47 ± 16	150	12	6
Kidneybeans (canned)	52	150	17	9
Lentils, green boiled	30 ± 4	150	17	5
Peanuts	14 ± 8	50	6	1
Bananas (raw)	52 ± 4	120	24	12
Grapes white (raw)	46 ± 3	120	18	8
Mango	51 ± 3	120	17	8
Watermelon	72 ± 13	120	6	4
Apples	38 ± 2	120	15	6
Oranges	42 ± 3	120	11	5
Peaches	42 ± 14	120	11	5
Strawberry (raw)	40 ± 7	120	3	1
Milk (full fat)	27 ± 4	250	12	3
Yoghurt (full fat)	36 ± 4	200	9	3
Ice cream	61 ± 7	50	13	8

Modified from [60]

GL restriction has several advantages: (a) Preclusion of hunger attacks between meals by hyperinsulinemia with relative hypoglycemia, (b) unlimited access to other macronutrients inducing improved satiety and maintenance of weight loss [55], (c) a reduction of cardiovascular risk and IR independent of weight loss [30••, 45••, 51, 52, 56, 62, 63], and (d) a reduction or abolishment of antidiabetic medication in line with preventing hypoglycemia [30••, 45••].This return to a healthier life increases patients’ compliance and minimizes the potentially ominous side effects of antidiabetics [64]. A GL restriction is now implemented in the guidelines for type-2 diabetes [65]. Substantial evidence supports its therapeutic use as safe and immediately effective [32••, 37•, 43, 50, 63, 66, 67].

(3) Reduction of Dyslipoproteinemia and Hypertension:

High triglyceride, VDLDL, LDL, and low HDL cholesterol levels characterize the atherogenic dyslipoproteinemia in CMS that may be improved by macronutrient modification without weight loss [34, 68]. Low-fat/high-carbohydrate nutrition effectively reduced LDL but deteriorated triglycerides, VDLDL, and HDL [69]. However, a partial replacement of carbohydrate content with an isocaloric amount of fat (preferably monounsaturated fatty acids (MUFA)) improved all factors of dyslipoproteinemia, confirmed in meta-analysis for healthy and diabetic individuals and the Mediterranean diet [44, 68, 70, 71]. Supplementation with omega-3-fatty acids may reduce serum triglycerides [72]. Given the risks of non-alcoholic fatty-liver disease in CMS, alcohol consumption should be low-to-moderate [34].

CMS associated hypertension may be treated by weight loss alone. Other options imply a restriction of sodium to about 4 g daily, or increases in the intake of potassium, calcium, long-chain omega-3 fatty acids and protein, in particular vegetable protein [34]. The Omni Heart Study (Optimal Macronutrient Intake Trial for Heart Health) successfully combined these factors subsequent to the DASH diet (dietary approach to stop hypertension) promoting the intake of vegetable, nuts, fruit, full grain, and lean milk products, poultry and fish [73, 74]. This macronutrient modification is consistent with the observed lowering of blood pressure via normalized hyperinsulinemia by GI/GL restriction [30••, 50, 62] also in the higher-fat DASH diet promoting full-fat dairy [75].

Of relevance, this nutritional modification with restricted carbohydrate intake and more protein and fat intake—referring particularly to vegetable protein and vegetable oil—is safe and lowers the risks for CAD and mortality [76].

#### Exercise Training in CMS

A recent meta-analysis confirmed that exercise has beneficial effects on triglycerides, HDL, apolipoprotein A1, leptin, interleukin-18, fibrinogen, and angiotensin II but not for C-reactive protein, interleukin-6, tumor necrosis factor α, or adiponectin [77]. The beneficial effects on insulin metabolism were substantially enlarged in the presence of CMS risk factors. Both moderate and vigorous exercise training improved cardiometabolic health [77].

Aerobic exercise is recommended for the sedentary beginner as walking [78] or biking with incrementally increasing duration and intensity along the measure of keeping a conversation ongoing. Resistance training has also shown beneficial effects on HbA1c, fat mass, and systolic blood pressure but not on triglycerides, cholesterol levels, and diastolic blood pressure in patients with dysglycemia [79]. A measurable reduction of mortality was found even for modest incremental increases of fitness and training [80]. Further studies are needed to explore the apparent superiority of aerobic training for risk factors in CMS and the interaction with nutritionally deteriorated IR.

#### Bariatric Surgery

Bariatric surgery implies gastric banding, sleeve gastrectomy, the Roux-en-Y gastric bypass, and biliopancreatic diversion techniques. Recently, the indication was extended from severe obesity to mild-to-moderate obesity combined with type-2 diabetes or CMS. Bariatric surgery may induce remission of type-2 diabetes in about 60 % after 6 years and 36 % after 10 years [81]. The immediate effects of glucose lowering stem from the sudden negative calorie balance and the increase of GLP-1 response on food delivery into the small intestine. Long-term effects are based on the excess weight loss with its increase in insulin sensitivity. The prevalence of CMS may be reduced by 60–95 % in 1–3 years [82] and that of hypertension and the other risk factors by >30 % [22••].

### Pharmacological Agents

From the three recent options for overweight treatment, orlistat, sibutramine, and rimonabant, only orlistat is currently available. It modestly lowers weight associated with improvements in LDL, fasting insulin, blood pressure, and potentially diastolic function [22••].

The pharmacological therapy of hypertension, of dyslipoproteinemia, and of diabetes is well established. Its discussion would be beyond the scope of this review that is focused on new developments in association with CMS and the risk of HF.

### Non-Pharmacologic Therapeutic Strategies for DD/HFPEF

Given the different cardiovascular comorbidities with their specific treatments, therapeutic studies in DD/HFPEF need specifically selected participants. The uncertainty about the triggers for a progress from DD to HFPEF makes the effective treatment of DD an important preventive intervention so that the discussion of DD and HFPEF treatment strategies is combined.

By now, promising alternative strategies exist for treating the underlying CMS. The respective clinical studies are rare for several reasons. Firstly, HF, DD, and cardiovascular risks are not routinely assessed in randomized trials on CMS. Secondly, studies on life style changes most commonly do not asses DD. Thirdly, research on nutrition and exercise is difficult to finance being not in the promotional scientific program of pharmaceutical industry whereas the beneficial potential for the affected patients may save millions of dollars annually for the public health systems.

#### Dietary Approaches

In obese type-2 diabetics, weight loss nutrition (460 kcal daily for 4 months) improved DD and myocardial triglyceride content [83]. In severe obesity, low GI diet vs. bariatric surgery both induced relevant weight loss and improved LV mass and DD after 1 year [84]. Moderate aerobic training was the exercise intervention for a comparison of low-fat/high-carbohydrate nutrition (1800 kcal) with an isocaloric GL restricted diet in obese type-2 diabetics (HbA1c 7,1 %) [30••] achieving similar reductions in weight, total cholesterol, and HbA1c after 3 weeks. Only GL restriction led to significant improvements of postprandial glucose and insulin levels, IR, triglycerides, blood pressure, diastolic function, and exercise capacity, i.e. all factors of CMS. In the parallel group on low-fat diet, these metabolic and functional parameters remained unchanged but improved in the subsequent crossover design with GL restriction for 2 weeks [30••]. These results were obtained in spite of a more than 50 % reduction of the antidiabetic medical therapy with GL restriction necessary to avoid hypoglycemia vs. 16 % with low-fat nutrition. This major reduction of antidiabetic medications implies that modification/restriction of carbohydrate intake is a causal therapy of CMS and its associated myocardial dysfunction [26•, 85]. Evidence confirms such a major reduction of antidiabetic medication subsequent to GL restriction in type-2 and type-1 diabetes both for oral antidiabetics and for insulin [45••, 50, 85].

Regarding dietary effects on HFPEF in CMS, a case report in an obese type-2 diabetic on insulin therapy with IR and a recent onset of HFPEF showed reversal of DD by GL restriction within 3 weeks but without relevant weight loss [66]. A study in “hypertensive” HFPEF in 13 individuals with treated hypertension and stable HFPEF demonstrated improvements of blood pressure, DD, and HFPEF symptoms by the low-sodium DASH diet [86] that implies also lowered GI/GL [72]. Considering that the study participants had a mean BMI of 35.5 ± 7.9 kg/m^2^ and that 43 % had type-2 diabetes, the etiology of HFPEF might have been regarded to relate both to IR and hypertensive heart disease but this issue was not discussed in line with no information provided regarding weight loss.

Summarizing, relevant weight loss or normalization of IR induced by GL restriction or bariatric surgery unequivocally improved DD [30••, 66, 83, 84, 86].

#### Exercise Training

In spite of the accepted beneficial effects of exercise for cardiovascular risk factors in CMS, the effects on the associated DD are ambiguous. Notably, the respective study designs for exercise in obese type-2 diabetics have maintained the traditional low-fat/high-carbohydrate nutrition [87, 88]. Their ambiguous outcome fits the results of the low-fat vs. GL restriction dietary arm in spite of moderate aerobic training as discussed above [30••]. A possible mechanism behind this apparent discrepancy of exercise effects has recently been shown: A single carbohydrate meal may worsen DD in type-2 diabetics and non-diabetic individuals [89•]. This suggests that the beneficial effects of exercise on DD may be neutralized by nutritional deterioration in IR.

Improved physical performance and DD by exercise training have been shown in HFPEF patients with overweight (BMI 31 ± 5 kg/m^2^) and additional ≥1 risk factors [31] but this report, like so many exercise studies, does not report the nutritional recommendations during the intervention.

A recent meta-analysis of six studies concluded that exercise in HFPEF does not improve DD in spite of improvements in cardiorespiratory fitness and quality of life [90]. However, the echocardiographic parameters used to assess DD relied on E/A and/or deceleration time that imply bimodal changes in the course of diastolic disease rendering the respective interpretation of interventional results difficult.

Summarizing these exercise studies, the assessment of diastolic function should use tissue Doppler derived and quantifiable methods of assessing DD [21••]. This diagnostic method should be applied for assessing the potential of IR normalization for the treatment/reversal of DD via both specific exercise and nutritional interventions.

### Pharmaceutical Therapy

Given the diverse comorbidities of DD/HFPEF, the respective treatment approach needs to primarily address this underlying problem. Concerning CAD, arterial hypertension, and peripheral vascular disease, the respective treatments are well established and beyond the scope of this review. Instead, discussion presents developments in the therapy of IR and type-2 diabetes with potential for improving DD/HF.

#### Insulin Resistance

The *biguanide* metformin improves hepatic and peripheral IR and LV diastolic function in patients with type-2 diabetes [91]. It affects translocation of glucose transporters Glut1 and Glut4, prevents hyperglycemia induced abnormalities in relaxation by reducing intracellular Ca transients, and maintains metabolic flexibility [22••].

*Glitazones* had been designed to increase insulin sensitivity. However, the associated effect on renal collecting ducts is fluid retention so that use of these drugs was not recommended for patients with (advanced) HF. In type-2 diabetics without cardiac disease, DD was improved by pioglitazone [92].

Within the *GPL-1 analogues*, liraglutide in the LEADER trial [93] has just been announced to effectively improve CV deaths but not systolic HF whereas lixisenatide did not alter cardiovascular events and HF hospitalization in patients with type-2 diabetes and acute coronary syndrome [94]. Studies on DD/HF in diabetes are underway.

Data for the protective action of *DDP-4 inhibitors* are inconclusive. Sitagliptin was shown to potentially improve DD [95]. In a large retrospective study, the use of DPP-4 inhibitors compared with sulphonylureas was associated with a reduced risk of hospitalization for HF whereas an earlier comparison of saxagliptin with placebo had demonstrated an increased risk for HF hospitalization [96].

The recent *sodium/glucose cotransporter-2 (SGLT2) inhibitor* empagliflozin lowers blood glucose irrespective of beta-cell function and IR by blocking glucose reabsorption in the proximal renal tubulus with subsequent excretion of abundant glucose. This elimination precludes protective metabolic mechanisms against hyperglycemic and insulinemic peaks. The EMPA-REG outcome study data showed a decrease of CV deaths and an improved hospitalization rate with HF [97•]. Studies on DD and HF in patients without and with type-2 diabetes are underway.

#### Glucose-Lowering Antidiabetic Therapy

Awareness has grown for the risks from hypoglycemic events and/or the simultaneous use of several antidiabetics [64]. Nevertheless, evidence shows that glucose control does matter in patients with diabetes [98], especially for the risk of DD/HF from postprandial dysmetabolism and IR [27, 99•]. Besides metformin, insulin therapy may be protective: multiple daily injection (MDI) vs. mixed insulin regimen and the use of analogue MDI vs. human insulin MDI regimen have shown specifically lowered postprandial glucose levels and improved myocardial function and perfusion [8, 99•, 100].

Unfortunately, the assessment of postprandial metabolism is ignored in most evidence creating studies. Furthermore, the different stages of IR deserve consideration for tailoring therapeutic strategies. Given the nutritional induction of postprandial hyperglycemia in IR and type-2 diabetes, carbohydrate restriction remains the most appropriate antidiabetic therapy.

Summarizing, patients with significant hyperglycemia benefit most from immediate insulin-based strategies that improve especially postprandial glucose, and those patients with stable glucose control should receive immediate carbohydrate restriction combined with meticulous adaptation of their individual antidiabetic regimen.

## Conclusion: Risk of DD/HFPEF in CMS

The diagnostic design of respective therapeutical studies in overt HFPEF or for its prevention is substantially and biomathematically improved by the new approach for quantification of DD via the age dependence of diastolic function [21••] and should apply a homogenous selection of CMS individuals in order to create the necessary statistical evidence.

Patients with CMS and DD have as underlying key mechanism depleted cardiac energetic reserve caused by IR. Given unequivocous clinical studies on the dietary treatment of DD/HFPEF and the consistent evidence from dietary treatment of the underlying CMS, causal therapy is a normalization of insulin sensitivity by the therapeutic use of diets with modified macronutrient composition: increased vegetables, fruits and salads, reduced GL in favor of full grain products, and liberalization of fat in favor of MUFA/PUFA associated with an aerobic exercise program.

## Abbreviations

CAD: Coronary artery disease
CMS: Cardiometabolic syndrome
DD: Diastolic dysfunction
HDL: High-density lipoprotein cholesterol
HF: Heart failure
IR: Insulin resistance
LDL: Low-density lipoprotein cholesterol
LV: Left ventricle
MUFA: Monounsaturated fatty acids
RAAS: Renin-angiotensin-aldosterone system
VDLDL: Very dense LDL particles
